# Sexual dysfunctions and sexual behaviors of normal weight, overweight and obese sexual minority men

**DOI:** 10.1192/j.eurpsy.2021.1465

**Published:** 2021-08-13

**Authors:** H. Pereira

**Affiliations:** 1 Ubi, Research Centre in Sports Sciences, Health Sciences and Human Development, Covilha, Portugal; 2 Cics-ubi, Centre for Research in Health Sciences, Covilha, Portugal; 3 Psychology And Education, University of Beira Interior, Covilha, Portugal

**Keywords:** sexual dysfunctions, Sexual Behavior, obesity, Gay

## Abstract

**Introduction:**

With the growing recognition of overweightness and obesity as significant, international public health concerns, the body of research investigating the relationship between body mass index (BMI), sexual health and sexual functioning in sexual minority men is still scarce.

**Objectives:**

The purpose of this study is to assess sexual health determinants (sexual behavior and sexual functioning) in relation to normal weight, overweightness and obesity among gay and bisexual men.

**Methods:**

The survey included four categories of questions/measurements, encompassing sociodemographic information, protected/unprotected sexual behaviors, sexual functioning, and BMI. The survey was conducted online, and recruitment consisted of online notifications (emails and electronic messages), and advertisements sent to LGBT community organizations, mailing lists, and social networks.

**Results:**

The study sample was composed of 741 gay and bisexual men, ranging in age from 21 to 75 years old (M_age_=43.30, SD_age_=11.37), 62.5% of men self-identified as gay and 37.5% as bisexual. Prevalence of normal weight was 50.3%, of overweight 33.3% and of obesity 16.4%. Hierarchical multiple regression analysis to assess the effects of BMI on sexual health showed that being younger in age, self-identifying as gay, being in a relationship, having longer penises, adopting insertive position in sex and being normal weight were significant predictors of anal receptive sex without condoms, explaining 24.2% of the total variance. Yet, BMI was not predictive of sexual functioning.

**Conclusions:**

These findings highlight the importance of including BMI in sexual behavior models of sexual minority men to better understand BMI’s role in influencing sexual risk.

